# Risk of Atrial Fibrillation and Stroke in Patients with Hypertrophic Obstructive Cardiomyopathy Treated by Modified Morrow Septal Myectomy: Reports of a Propensity Score Matching Cohort

**DOI:** 10.3390/jcdd12090321

**Published:** 2025-08-22

**Authors:** Yi-Xi Zou, Xi-Lin Zhang, Jian-Peng Zheng, Feng Lu, Gregory Y. H. Lip, Ying Bai, Yu-Feng Sun, Wei-Hua Guo

**Affiliations:** 1 Department of Cardiac Surgery, Beijing Anzhen Hospital, Capital Medical University, Beijing 100029, China; 2 Cardiovascular Center, Beijing Tongren Hospital, Capital Medical University, Beijing 100176, China; 3 Beijing Municipal Health Commission Information Center, Beijing 100054, China; 4 Liverpool Centre for Cardiovascular Science at University of Liverpool, Liverpool John Moores Universtiy and Liverpool Heart & Chest Hospital, Liverpool L69 3BX, UK; gregory.lip@liverpool.ac.uk; 5 Department of Clinical Medicine, Aalborg University, 7K 9220 Aalborg, Denmark; 6 Clinical Medical Laboratory, Beijing Tongren Hospital, Capital Medical University, Beijing 100176, China; 7 Dongzhimen Hospital, Beijing University of Chinese Medicine, Beijing 101121, China

**Keywords:** modified Morrow septal myectomy, obstructive hypertrophic cardiomyopathy, atrial fibrillation, stroke

## Abstract

Background: A modified Morrow septal myectomy (MMSM) is one of the main treatment methods for obstructive hypertrophic cardiomyopathy (OHCM). Our aim was to study the impact of MMSM on the risk of AF and stroke in OHCM patients. Methods and Results: From 1 January 2014 to 31 December 2020, 6426 patients with obstructive HCM (OHCM) were selected from the Beijing Municipal Health Commission Information Center (BMHCIC) datasets (mean age: 54.3 years; 43.8% female). After propensity score matching, 3780 patients were selected, including 1890 who received MMSM (Group 1) and 1890 who did not receive any surgery (Group 2). During a median of 0.8 (interquartile range [IQR]: 0.1, 2.7) years of follow-up after discharge from the hospital, stroke risk was lower in Group 1 compared to Group 2 (aHR: 0.4, 95%CI: 0.2–0.6, *p* < 0.001), and the results were further confirmed by Kaplan–Meier analyses (*p* < 0.001). There was no statistically significant difference in the risk of AF (aHR: 1.0, 95%CI: 0.7–1.5, *p* = 0.991). The risk of AF decreased in the first 6 years since receiving MMSM and then sharply increased beyond 6 years after MMSM according to Kaplan–Meier analyses. Conclusions: MMSM is associated with a decreased risk of stroke in OHCM patients. The risk of AF decreased in the first 6 years since receiving MMSM and then sharply increased beyond 6 years after MMSM.

## 1. Introduction

Hypertrophic cardiomyopathy (HCM) is a complex and relatively common genetic cardiac disease characterized by increased left ventricular (LV) wall thickness that is not solely explained by abnormal loading conditions [1,2]. According to different structural and functional changes, HCM can be divided into three types: obstructive hypertrophic cardiomyopathy (OHCM) with obvious left ventricular outflow tract obstruction (LVOTO), non-obstructive hypertrophic cardiomyopathy (N-OHCM), and apical hypertrophic cardiomyopathy (AHCM) [2,3]. Almost two-thirds of HCM patients suffer from the obstructive condition. [4]. LVOTO is defined as a peak instantaneous Doppler LV outflow tract gradient of ≥30 mmHg, with outcomes such as mitral regurgitation, arrhythmia, congestive heart failure, and sudden cardiac death (SCD) [2].

Nowadays, medication, alcohol ablation, or septal myectomy surgery, such as classic Morrow surgery, is usually used to improve quality of life in OHCM patients [2,5,6].

For example, mavacamten is a cardiac myosin inhibitor, which could improve exercise capacity, LVOTO, New York Heart Association (NYHA) functional class, and health status in patients with OHCM [7,8]. While the mean reduction in the left ventricular ejection fraction (LVEF) was just 4%, 7 of the 123 (5.7%) patients treated with mavacamten in EXPLORER-HCM had an episode of transient systolic dysfunction (LVEF, <50%) over 30 weeks of treatment [9]. The transapical myocardial revascularization system is an emerging technology that holds great promise as a surgical approach for hypertrophic cardiomyopathy in the future. However, the indications are relatively narrow due to the limited sample size and the ambiguous medium-term and long-term effects, especially the possible side effects of the left bundle branch block [10].

Among all the available treatments, Morrow surgery is currently considered as the gold standard for relieving LVOTO in both adults and children with OHCM. Patients are considered as candidates for surgery if they have obvious outflow gradients measured by continues wave Doppler echocardiography or present with exertional dyspnea, chest pain, and categories III or IV in NYHA functional classes [11]. After surgical myectomy, obstruction and serious complications are significantly reduced. With over forty years of development, septal resection, also known as modified Morrow septal myectomy (MMSM), is now extended or combined with mitral valve replacement or anterior mitral leaflet plication in order to reduce or eliminate LVOTO [12]. In addition, MMSM has also been effective in improving symptoms in the long term [13].

Atrial fibrillation (AF) is the most common arrhythmia complicating HCM with an incidence ranging from 22% to 32% [14,15,16]. Patients with OHCM have a high risk of AF and stroke [17]. The pathophysiological and anatomical changes between AF and HCM are complex. According to the existing research, AF in OHCM patients is usually caused by higher left ventricular pressure and elevated left atrial pressure due to the thick myocardium and reduced diastolic relaxation. Because MMSM could reduce the left ventricular outflow tract, we suspected that the incidence of AF in OHCM patients could accordingly be reduced after MMSM. In addition, because AF is one of the main reasons leading to stroke, whether the risk of stroke could be correspondingly changed with AF risk change needs further exploration.

## 2. Methods

The study protocol conformed to the ethical guidelines of the Declaration of Helsinki and was approved by the Ethical committee of Beijing Tongren Hospital, Capital Medical University (No.: TREC2022-KY011; Date: 31 March 2022). Informed consent was waived due to anonymized and unidentified information being used for the retrospective analysis.

The study selected OHCM patients in the Beijing Municipal Health Commission Information Center (BMHCIC) database of China from 1 January 2014 to 31 December 2020. BMHCIC is a mandatory health surveillance and supervision government agency requiring medical information to be uploaded from all 153 hospitals/centers located in the overall Beijing area. The OHCM patients were selected from BMHCIC as previously described [18]. All patients’ in-hospital records and outpatient records were stored in the BMHCIC. The quality of the medical records was validated by the BMHCIC through inspection and improvement plans. 

The patients received electrocardiogram (ECG) first, and then echocardiography or cardiac magnetic resonance imaging was performed to diagnose OHCM according to the recommendations of the AHA. Their diagnoses were stored using the claims for diagnostic codes *(International Classification of Diseases, Tenth Revision, Clinical Modification; ICD-10-CM)* with I42.101 for OHCM [19]. Only patients with the first diagnosis of OHCM in a hospital setting were included in this study. The patients were excluded if they were treated through mitral valve treatment or mitral valve repair but without MMSM treatment.

The included patients were divided into two groups, namely patients with OHCM treated by MMSM (Group 1) and patients with OHCM without any surgical treatment (Group 2).

Diagnosis of AF was established according to patients’ ECG recordings or 24-h Holter monitoring by the cardiologists and stored with *ICD-10-CM* codes of I48. Diagnosis of stroke was established according to patients’ symptoms and related imaging examinations by neurologists and radiologists and stored using *ICD-10-CM* codes of I63, including ischemic stroke and hemorrhagic stroke. All the diagnoses were built up at the time when the patients were discharged from the hospital or had their outpatient visits. And these diagnoses and dates were confirmed through reviewing medical records. Echocardiographic parameters were obtained from medical recordings at enrollment or after the Morrow procedure. First-time diagnosis with HCM or HCM complicated by AF and stroke was considered as the date of onset for the respective conditions.

Clinical data, including age, sex, history of stroke, hypertension, New York Heart Association (NYHA) Function Classification, and clinical outcomes, were extracted from the BMHCIC database and rechecked by reading their medical records. The follow-up date was the date of their discharge. Follow-up information after first discharge was obtained from BMHCIC outpatient records and readmission in-hospital records. All the patients’ in-hospital records and outpatient records could be obtained from all over the Beijing District and were connected with their medical insurance numbers. The last recorded visit date or the end of the study (31 December 2020) was considered as the end of the follow-up. The information on AF and stroke during follow-up was obtained through their ICD codes of medical records. The follow-up ended when the first times these events occurred were obtained.

Modified Morrow Septal Myectomy (MMSM) is a procedure performed under general anesthesia with low-temperature extracorporeal circulation, with continuous anterograde cold blood cardioplegia used for myocardial protection. Operations were guided by transthoracic echocardiography and transesophageal echocardiography. For the modified Morrow procedure, the incision was made by extending the classic incision with a midventricular resection; continued resection was made leftward toward the mitral valve annulus and apically to the bases of the septum or ventricular free wall, and anomalous chordal structures, muscle bundles, and fibrous attachments of the mitral leaflets to the ventricular septum or free wall were divided or excised [12]. This procedure is coded using the International Classification of Diseases, Ninth Revision, Clinical Modification *(ICD-9-CM)* codes of 37.35002. If the patients had AF, they might receive MAZE with *ICD-9-CM* codes of 37.33002 and atrial appendage ligation (AAL) with *ICD-9-CM* codes of 37.49006 during MMSM.

### Statistical Analysis

Data were expressed using the mean ± SD for continuous variables and number (percentage %) for categorical variables. Continuous and categorical variables were compared using an ANOVA test and *t* test, respectively. New-onset AF and stroke events after discharge periods were calculated. To reduce the impact of selection bias, baseline differences in OHCM patients were adjusted by propensity score matching, which was based on 1:1 matching within a prespecified caliper width and without replacement. Echocardiographic parameters were compared before and after the Morrow procedure with the chi-squared test for categorical variables and with a paired T test for continuous variables. The incidence of AF or stroke of patients after discharge was evaluated with Kaplan–Meier curves. *p* < 0.05 was considered as statistically significant. All statistical analyses were performed using the SPSS 24.0 software.

## 3. Results

In total, 6426 patients diagnosed with OHCM were selected from the BMHCIC datasets (mean age: 54.3 years; 43.8% female). Of those, 2438 received MMSM and 3988 did not receive any surgery. After propensity score matching, 1890 received MMSM (Group 1) and 1890 did not receive any surgery (Group 2). Baseline characteristics before and after propensity score matching are shown in Table 1. The echocardiographic parameters before and after MMSM were compared and are shown in Table 2.

### 3.1. Atrial Fibrillation

During a median follow-up of 0.8 (interquartile [IQR]: 0.1, 2.7) years, 132 (3.4%) patients were diagnosed with new-onset AF after discharge, as shown in Table 3. Cox regression analysis was used for exploration of the risk of AF after discharge. And there was no statistically significant difference between Group 1 and Group 2 (aHR: 1.0, 95% CI: 0.7–1.5, *p* = 0.991).

**Figure 1:**
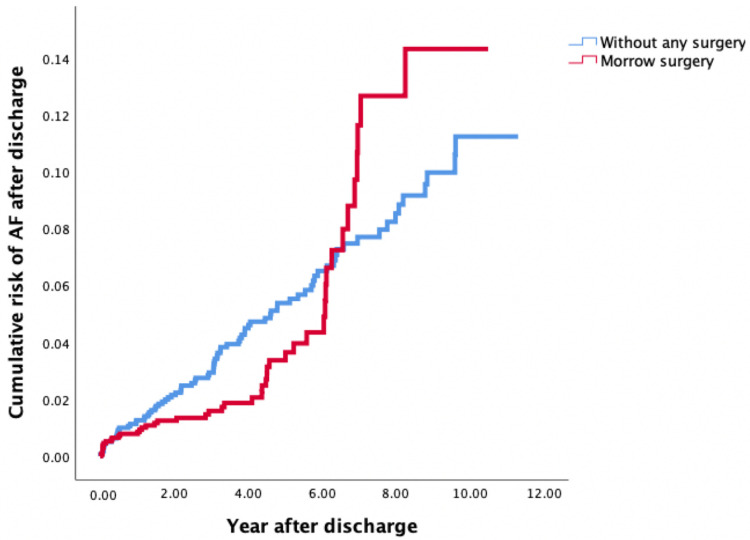
Risk of AF after discharge among patients with OHCM treated by MMSM (Group 1) and OHCM patients without any surgical treatment (Group 2) according to Kaplan–Meier analysis. AF, atrial fibrillation; OHCM, obstructive hypertrophic cardiomyopathy; MMSM, modified Morrow septal myectomy.

According to the Kaplan–Meier analysis, the risk of AF was lower in patients receiving MMSM in the first 6 years, and this risk sharply increased 6 years after MMSM (log-rank test: *p* = 0.387) (Figure 1).

### 3.2. Stroke and Mortality

**Figure 2:**
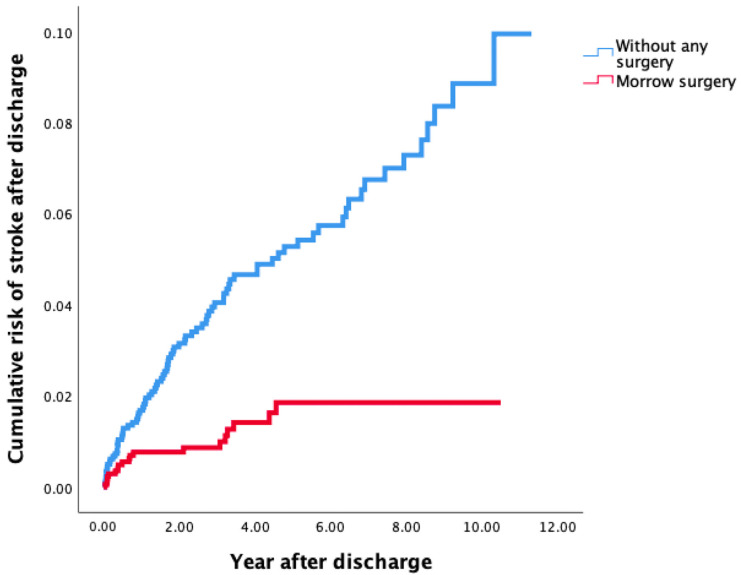
Risk of stroke after discharge among patients with OHCM treated by MMSM (Group 1) and in OHCM patients without MMSM (Group 2) according to Kaplan–Meier analysis. OHCM, obstructive hypertrophic cardiomyopathy; MMSM, modified Morrow septal myectomy.

As shown in Table 3, 103 (2.7%) patients were diagnosed with new stroke after discharge with a median follow-up of 0.8 (interquartile [IQR]: 0.1, 2.7) years. Of these stroke patients, 10 patients had hemorrhagic stroke before propensity score matching, only 1 patient with hemorrhagic stroke was included without Morrow surgery, and 0 patient with hemorrhage was included with Morrow surgery after propensity score matching. Compared to Group 2, Group 1 was associated with a decreased risk of stroke according to Cox regression analysis. (aHR: 0.4, 95%CI: 0.2–0.6, *p* < 0.001). The results were further confirmed by Kaplan–Meier analysis with log-rank tests (*p* < 0.001) (Figure 2).

## 4. Discussion

In the current study of OHCM, MMSM is associated with a decreased risk of stroke, but AF risk decreased in the first 6 years since performing MMSM and then sharply increased beyond 6 years after MMSM.

AF is a common arrhythmia in HCM patients, leading to an increased risk of morbidity and mortality, especially in patients with obstructive left outflow tract [16,20]. The frequency of AF with HCM increased by approximately five times [21]. The pathophysiology of AF in patients with HCM is complex. The main mechanism is the elevated left atrial pressure caused by increased ventricular filling pressure due to the thick myocardium, especially in patients with LVOTO [22], whose LV filling mainly relies on LA contraction [23]. In the classic Morrow procedure, a reduction in LVOTO is usually achieved by removing 5–10 g of myocardium from the basal interventricular septum. Recently, MMSM with the aim of dredging left ventricular outflow by extending the resected area was reported with sustained effects of relieving symptoms and eliminating Systolic Anterior Motion (SAM) [10,24]. In our study, the thickness of the myocardium was reduced by MMSM, and mitral valve insufficiency was amended according to the echocardiographic reports before and after the procedure. Therefore, we suspected that the risk of AF and AF-related stroke could be reduced by procedures reducing LVOTO, such as MMSM. Unfortunately, the risk of new-onset AF sharply increased 6 years after MMSM due to the recurrence of OHCM. Previous AF could possibly be relieved by MAZE, which is usually performed in those who have AF history at enrollment. But Morrow seemed to have little impact on new AF; therefore, the long-term risk of AF was not accordingly reduced. A previous study showed a similar trend of AF risk after septal myectomy with an increasing length of follow-up.

In our study, the risk of stroke was strongly reduced by MMSM in OHCM patients compared to those without any surgery, as was our expectation. But the risk of stroke did not show a similar trend to the risk of AF, though AF in HCM conferred an eight times increased stroke risk [25]. Apparently, our study strongly confirmed the benefits of MMSM surgery by reducing the risk of stroke in OHCM patients. Other short-term or long-term effects of MMSM surgery were also reported in previous studies [12,26], but there is little information on the risk of AF and stroke. This was the first study to show the relationship between MMSM surgery and the risk of AF and stroke.

Of the patients with HCM who had an ischemic stroke, 67% had concurrent AF in a study from Japan [27]. As such, AF was associated with 8-fold higher risk of stroke and thromboembolism in HCM patients than those in sinus rhythm. However, in the study of Shintaro Haruki et al. involving 593 patients with HCM, 11.5% experienced stroke and systemic embolic events, with this number decreasing to 9.0% after excluding patients with previously documented AF, which convinced us that HCM was an independent risk of stroke. They also reported that an enlarged LAD was an independent determinant of stroke and systemic embolic events in patients without previously documented AF [28]. The residual risk of stroke related to pre-existed AF could be reduced through AAL during the protocol which increased the clinical meaningfulness of the current study. Some recent studies confirmed similar stroke rates between patients with AF and SR [29]. Though a beneficial effect of AAL during surgery was observed, there was insufficient evidence to support AAL in addition to heart surgery to protect against thromboembolisms related to HCM. Therefore, the necessity of concomitant AAL in OHCM patients without baseline AF but at high risk of developing postoperative AF remains unclear and warrants extensive future investigation.

Postoperative stroke risk was not parallel to AF risk in OHCM patients even if they had MMSM surgery, perhaps because the potential risk factors for stroke in young patients mainly include cardiac embolism and cryptogenic causes, and these factors would evolve into large artery atherosclerosis and lacunar with increasing age [30]. MMSM significantly reduced the risk of stroke in patients with OHCM compared to those without any surgery treatment. MMSM maintained cerebral perfusion and reduced the occurrence of ischemic stroke through relieving outflow obstruction and stabilizing cardiac hemodynamics. Furthermore, the relief of outflow obstruction and correction of mitral regurgitation could reduce blood stasis in the atrium and ventricle, which then decreased the formation of atrial and ventricular thrombi and consequently reduced the occurrence of stroke regardless of whether they had recurrent AF or new AF. In addition, AAL could effectively reduce the risk of stroke after MMSM, which possibly explains the decreased risk of stroke even if AF risk increased after 6 years.

## 5. Limitations

Limitations should be mentioned. First, like most studies on HCM, this was a multi-center retrospective study with common limitations inherent in this type of study. Second, clinical backgrounds of the two groups were different from each other, but propensity score matching was used to build two groups which had similar factors affecting the risk of AF and stroke. Third, as this was a retrospective analysis of the BMHCIC database, the selection of the disease names and the procedure names largely depended on ICD codes, which were built according to the physician reports of the joined hospitals. But we confirmed the diagnosis through reviewing detailed echocardiographic data, which increased the robustness of the study. Fourth, OHCM patients without MMSM might undergo a different procedure (e.g., alcohol ablation); therefore, this study suggests a benefit of this protocol of MMSM in reducing the risk of AF and stroke. Though the benefits of relief of outflow obstruction appears to be well-known, the novelty of this study was in establishing its advantages and disadvantages in exploring the risk of AF and stroke associated with MMSM. Fifth, the CHA2DS2-VASc score was not listed as a baseline characteristic because HCM was suggested to play a role in anticoagulation independent of the CHA2DS2-VASc score. AF type and some echocardiographic data, such as left atrial diameter (LAD), maximum velocity of left ventricular outflow tract (LVOT-MV), maximum velocity of left ventricular outflow tract, and left ventricular ejection fraction (LVEF) were sometimes missing for those without MMSM surgery treatment in this retrospective dataset. Therefore, these data were not used for baseline characteristics to build propensity score matching. Sixth, because the dataset was mainly derived from medical records in the capital of our country with well-performed routine physical examinations, this reduced the risk of bias in the results due to uneven follow-up between the patients with and without surgery. Seventh, anti-arrhythmic drug distribution in each group could not be obtained, and it would have interfered with the results.

## 6. Conclusions

In the current study of OHCM, MMSM is associated with a decreased risk of stroke, but AF risk decreased in the first 6 years since MMSM was performed and then sharply increased beyond 6 years after MMSM. This approach should be used in future large prospective clinical studies.

## Figures and Tables

**Table 1:** Baseline characteristics of the included patients.

	Before Matching			After Matching		
Variables	Group 1	Group 2	*p* Value	Group 1	Group 2	*p* Value
Number	2438	3988		1890	1890	
Age	46.7 ± 15.3	58.9 ± 16.1	<0.001	49.8 ± 13.9	50.6 ± 16.6	0.104
Female, n (%)	998 (40.9)	1818 (45.6)	<0.001	776 (41.1)	761 (40.3)	0.643
Hypertension, n (%)	652 (26.7)	2306 (57.8)	<0.001	622 (32.9)	637 (33.7)	0.629
Diabetes mellitus, n (%)	121 (5.0)	665 (16.7)	<0.001	118 (6.2)	128 (6.8)	0.553
Stroke/TIA before discharge, n (%)	49 (2.0)	326 (8.2)	<0.001	48 (2.5)	47 (2.5)	1.000
OMI, n (%)	349 (14.3)	1345 (33.7)	<0.001	343 (18.1)	324 (17.1)	0.442
LBBB, n (%)	15 (0.6)	23 (0.6)	0.868	15 (0.8)	11 (0.6)	0.556
PCI/CABG, n (%)	27 (1.1)	146 (3.7)	<0.001	27 (1.4)	24 (1.3)	0.778
III-degree AVB, n (%)	39 (1.6)	61 (1.5)	0.826	32 (1.7)	25 (1.3)	0.424
NYHA III/IV, n (%)	602 (24.7)	474 (11.9)	<0.001	319 (16.9)	330 (17.5)	0.666
Pacemaker implantation, n (%)	16 (0.7)	97 (2.4)	<0.001	16 (0.8)	10 (0.5)	0.325
Anticoagulation, n (%)	66 (2.7)	126 (3.2)	1.0	58 (3.1)	56 (3.0)	0.924

Group 1: patients with OHCM treated with MMSM; Group 2: patients with OHCM without any surgical treatment; MMSM: modified Morrow septal myectomy; OHCM: obstructive hypertrophic cardiomyopathy; OMI: old myocardial infarction; LBBB: left bundle branch block; NYHA class: New York Heart Function Association class.

**Table 2:** Echocardiographic parameters before and after modified Morrow septal myectomy.

Parameters	Before	After	*p* Value
Left Atrial Diameter, cm	41.4 ± 7.5	37.0 ± 8.3	<0.001
Left Ventricular End-Diastolic Diameter, cm	41.3 ± 5.1	40.5 ± 6.1	<0.001
LVEF, %	55.5 ± 3.3	62.8 ± 3.6	<0.001
Myocardium Thickness, cm	19.2 ± 6.3	15.6 ± 7.6	<0.001
Severe Mitral Insufficiency, n (%)	810.0 (42.8)	0.0 (0.0)	<0.001
Systolic Mitral Regurgitation Bundle Area, cm^2^	7.0 ± 3.3	3.5 ± 0.8	<0.001
LVOT-MV, cm/s	302.4 ± 233.7	157.3 ± 72.6	<0.001

LVOT-MV, maximum velocity of left ventricular outflow tract; LVEF, left ventricular ejection fraction.

**Table 3:** Risk of new AF and stroke after discharge.

	N, %	Univariate		Age-Adjusted		Sex-Adjusted		Multivariate	
		HR (95% CI)	*p* Value	HR (95% CI)	*p* Value	HR (95% CI)	*p* Value	HR (95% CI)	*p* Value
Before Matching									
AF									
Group 1	53, 2.2%	0.6 (0.4, 0.8)	0.001	1.0(0.7, 1.4)	1.0	0.6(0.4, 0.8)	0.001	1.0(0.7, 1.4)	0.914
Group 2	209, 5.2%	1		1		1		1	
Stroke									
Group 1	21, 0.9%	0.1 (0.1, 0.2)	<0.001	0.3(0.2, 0.5)	<0.001	0.1(0.1, 0.2)	<0.001	0.4(0.2, 0.6)	<0.001
Group 2	310, 7.8%	1		1		1		1	
After Matching									
AF									
Group 1	46, 2.4%	0.9 (0.6, 1.2)	0.387	1.01(0.7, 1.5)	0.959	0.85(0.6, 1.2)	0.391	1.0(0.7, 1.5)	0.991
Group 2	86, 4.6%	1		1		1		1	
Stroke									
Group 1	19, 1.0%	0.3 (0.2, 0.5)	<0.001	0.4(0.2, 0.7)	<0.001	0.3(0.2, 0.5)	<0.001	0.4(0.2, 0.6)	<0.001
Group 2	84, 4.4%	1		1	1			1	

Group 1: OHCM patients treated with MMSM; Group 2: OHCM patients without any surgical treatment; MMSM: modified Morrow septal myectomy; OHCM: obstructive hypertrophic cardiomyopathy.

## Data Availability

Related data and materials are available from the corresponding authors upon reasonable request.
